# Position- and posture-dependent vascular imaging—a scoping review

**DOI:** 10.1007/s00330-023-10154-9

**Published:** 2023-09-06

**Authors:** Jordy K. van Zandwijk, Jaimy A. Simmering, Richte C.L. Schuurmann, Frank F.J. Simonis, Bennie ten Haken, Jean-Paul P.M. de Vries, Robert H. Geelkerken

**Affiliations:** 1 Division of Vascular Surgery, Department of Surgery, Medisch Spectrum Twente, Enschede, The Netherlands; 2 Magnetic Detection & Imaging, Faculty of Science and Technology, Technical Medical Centre, University of Twente, Enschede, The Netherlands; 3 Multi-modality Medical Imaging (M3i) Group, Faculty of Science and Technology, Technical Medical Centre, University of Twente, Enschede, The Netherlands; 4 Division of Vascular Surgery, Department of Surgery, University Medical Center Groningen, Groningen, The Netherlands

**Keywords:** Diagnostic imaging, Posture, Blood vessels, Humans

## Abstract

**Objectives:**

Position- and posture-dependent deformation of the vascular system is a relatively unexplored field. The goal of this scoping review was to create an overview of existing vascular imaging modalities in different body positions and postures and address the subsequent changes in vascular anatomy.

**Methods:**

Scopus, Medline, and Cochrane were searched for literature published between January 1, 2000, and June 30, 2022, incorporating the following categories: image modality, anatomy, orientation, and outcomes.

**Results:**

Out of 2446 screened articles, we included 108. The majority of papers used ultrasound (US, *n* = 74) in different body positions and postures with diameter and cross-sectional area (CSA) as outcome measures. Magnetic resonance imaging (*n* = 22) and computed tomography (*n* = 8) were less frequently used but allowed for investigation of other geometrical measures such as vessel curvature and length. The venous system proved more sensitive to postural changes than the arterial system, which was seen as increasing diameters of veins below the level of the heart when going from supine to prone to standing positions, and vice versa.

**Conclusions:**

The influence of body positions and postures on vasculature was predominantly explored with US for vessel diameter and CSA. Posture-induced deformation and additional geometrical features that may be of interest for the (endovascular) treatment of vascular pathologies have been limitedly reported, such as length and curvature of an atherosclerotic popliteal artery during bending of the knee after stent placement. The most important clinical implications of positional changes are found in diagnosis, surgical planning, and follow-up after stent placement.

**Clinical relevance statement:**

This scoping review presents the current state and opportunities of position- and posture-dependent imaging of vascular structures using various imaging modalities that are relevant in the fields of clinical diagnosis, surgical planning, and follow-up after stent placement.

**Key Points:**

*• The influence of body positions and postures on the vasculature was predominantly investigated with US for vessel diameter and cross-sectional area.*

*• Research into geometrical deformation, such as vessel length and curvature adaptation, that may be of interest for the (endovascular) treatment of vascular pathologies is limited in different positions and postures.*

*• The most important clinical implications of postural changes are found in diagnosis, surgical planning, and follow-up after stent placement.*

**Supplementary information:**

The online version contains supplementary material available at 10.1007/s00330-023-10154-9.

## Introduction

Medical imaging modalities have been used for decades to visualize human vasculature. The most commonly used modalities are digital subtraction angiography (DSA), computed tomography angiography (CTA), magnetic resonance angiography (MRA), and ultrasound (US) [1, 2]. Vascular imaging is used to provide insights in anatomy, geometry, patency of blood vessels, and various vascular pathologies. Each modality has benefits and drawbacks in terms of spatial resolution, imaging speed, tissue contrast, field of view, reproducibility, cost, and safety.

Most often, the patient’s vascular anatomy is visualized in supine position with extended limbs. In some cases, however, clinical symptoms may be position or posture dependent. Therefore, the underlying vascular pathology will not always be visualized appropriately in supine position. The geometry (diameter, cross-sectional area, curvature, torsion, etc.) of vessels in the human body may differ in the supine, prone, and standing position, but also in flexed and/or rotated head and limb postures, which may be associated with clinical outcomes such as stenosis development [3]. When only visualizing the vascular structures in supine, neutral position, features predisposing vascular pathologies may be missed [4].

Some imaging modalities have already been clinically applied to visualize vasculature in body positions and postures other than a neutral supine position. An example is US in a posture with abducted arms, where the elevated limbs induce compression of arterial and/or venous structures, also referred to as thoracic outlet syndrome (TOS) [5]. Opportunities to visualize vasculature in an upright fashion have emerged in CT and MRI, but are often only applied in research settings and not commonly applied in clinical practice yet [6–8]. Still, postural and positional information can aid in several clinical situations, such as diagnosing posture-triggered atrial fibrillation or Bow Hunter’s syndrome [9–11], screw placement in prone body position during spine surgery without aortic injury [12], or in quantifying iliac artery deformation by musculoskeletal motion for the purpose of decreasing stent-graft-related complications [13]. Therefore, the potential and added value of vascular imaging in different body positions and posture remain largely unexplored. The goal of this scoping review was to obtain an overview of existing vascular imaging modalities in different body positions and postures and to address subsequent geometrical changes in vascular anatomy.

## Methods

This literature review complied with the Preferred Reporting Items for Systematic Reviews and Meta-Analyses (PRISMA) statement standards [14]. The review protocol was prospectively registered in the PROSPERO database (identifier CRD42021264322).

### Search strategy

A search in the databases Scopus, Medline, and Cochrane for literature published between January 1, 2000, and June 30, 2022, was performed. The search incorporated the following categories: (1) image modality, (2) anatomy, (3) orientation, and (4) outcomes. The exact search terms can be found in Table 1. To accommodate the different body positions and postures, subcategories were made for the orientation category: 3a represents body positions such as upright position; the changes in body postures such as flexion and extension (3b) of different body parts such as the elbow and knee (3c). The search terms within the categories were separated by Boolean OR, and the categories were separated by Boolean AND. Incorporation of the different subcategories for (3) orientation is presented in the last row of Table 1. An asterisk (*) was used to indicate a conjugation of the search term. No language restrictions were applied. After the elimination of double hits, two authors ((J.v.Z. and J.S.)) independently screened the search results on title and abstract to adhere to the inclusion criteria:

**Table 1:**
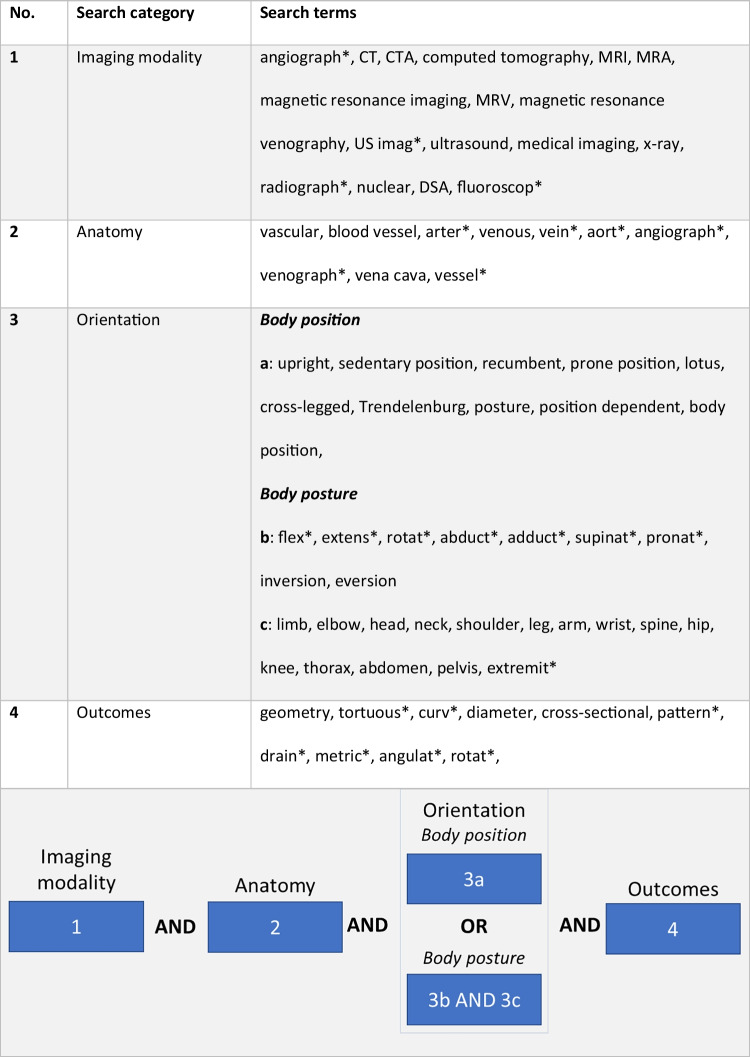
The categories and search terms used. The block diagram below the table shows the composition between categories with special attention to the orientation category that consisted of body position and body posture terms

1. Medical imaging was applied.

2. The study involved adult human subjects.

3. The anatomical focus was in the vascular domain.

4. The different position/posture was performed *during* imaging.

5. The different position/posture was compared to a standard reference position/posture.

6. Changes in outcome were stated as objective measure (measurable unit).

7. It was an original research article.

### Data extraction and quality assessment

For each study, the authors, publication year, subjects, vascular system, anatomical location, imaging modality, body posture/position, and study outcome measures were collected. Subsequently, each article was classified by the anatomical location: (1) head and neck, (2) thoracic, (3) abdominal, and (4) peripheral (arms and legs). Then, the data was sorted per vascular system that was being researched: (1) arterial, (2) venous, or (3) arterial and venous (AV). Additionally, the found studies were described while taking into account the different imaging modalities: (1) US, (2) CT, (3) MRI, (4) DSA, and (5) a combination of two or more (multiple) imaging modalities (MMOD). In case a large number of articles (≥ 20) was found on a specific disease or condition, these articles were excluded on eligibility at the authors’ discretion with the justification that a specialized review on that topic would be more appropriate.

To standardize the subject populations, we distinguished healthy volunteers from patients, where the type of patients or pathology was specified. To obtain consistency in position and posture descriptions, the following positional terms were used: supine, prone, lateral (also including lateral decubitus, recumbent, or park bench position), Trendelenburg (TB, also including head-down tilt), and reverse Trendelenburg (R-TB, also including head-up tilt). To focus on how blood vessels are affected by different positions and postures, the outcomes of the studies were reported in a generic way describing the observed trends without specific details (e.g., exact values, percentage changes, significance levels) for all studies and without replacing the original outcome term (e.g., diameter, CSA).

## Results

**Fig. 1:**
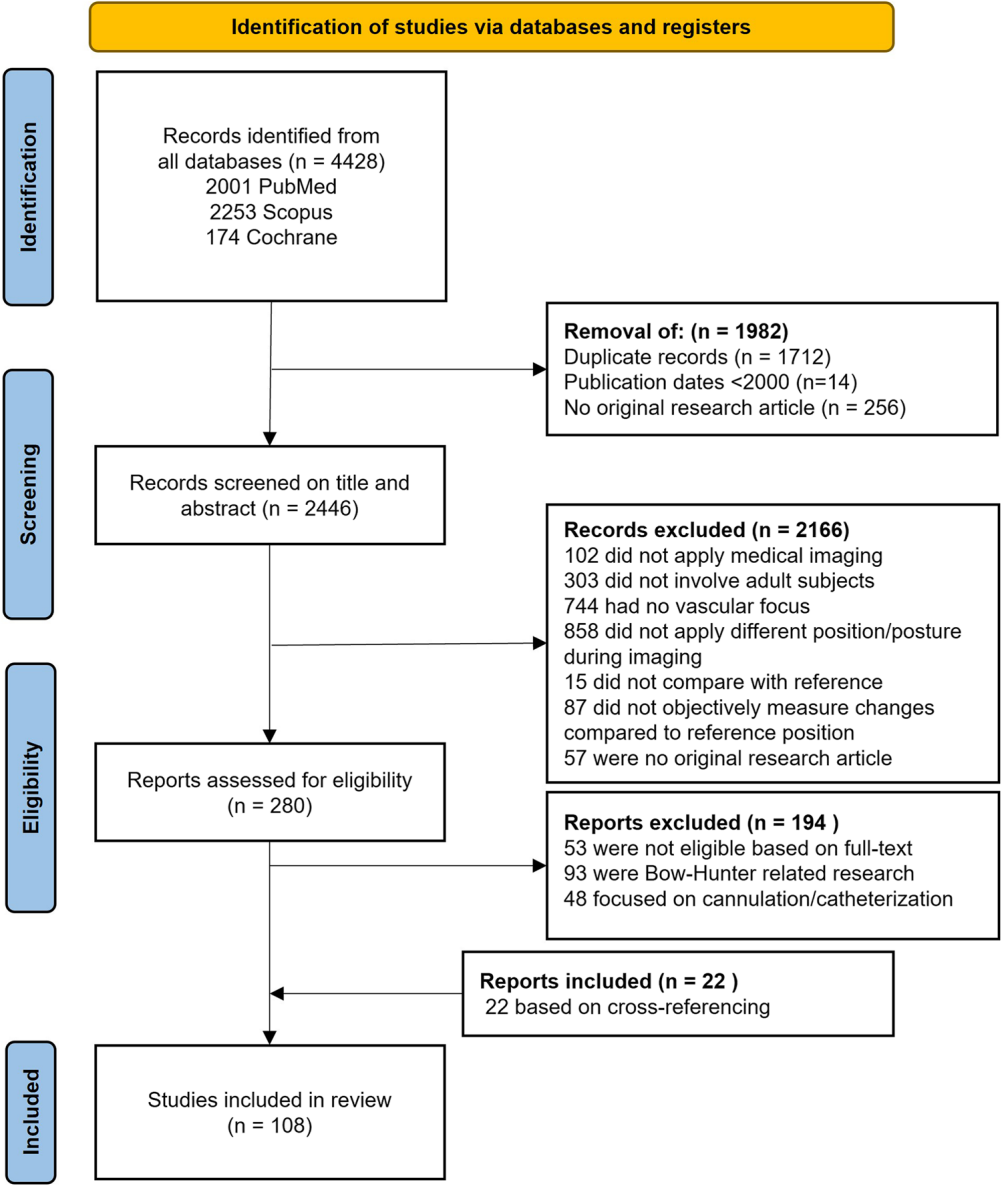
Flow chart of the studies included in the review

**Fig. 2:**
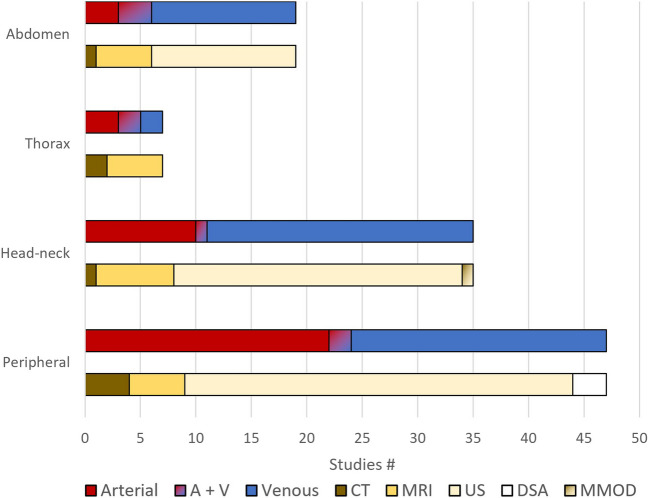
Included studies categorized by location and divided by the vascular system and modality and imaging modality. A+V = arterial and venous; CT = computed tomography; DSA = digital subtraction angiography; MMOD = multiple imaging modalities; MRI = magnetic resonance imaging; US = ultrasound

A total of 2446 articles were screened for adherence to the inclusion criteria, resulting in 280 articles that were evaluated for eligibility. A large part of these articles focused on the Bow Hunter’s syndrome (*n* = 93) and vessel dimensions in the scope of cannulation (*n* = 48). Because these are specific and widely investigated subjects, the authors consider them stand-alone topics that may overshadow other interesting findings of the present review and the corresponding articles were therefore deemed not eligible. Figure 1 illustrates the flowchart of the study selection. Characteristics of the included articles are shown in Table 2, categorized by anatomical location and imaging modality. The distribution of included articles over the different anatomical locations is shown in Fig. 2.

**Table 2:** Overview of studies included in this review

	Year	First author	Modality	System	Vessel(s)	Subjects	Position/posture	Outcome measures
Abdomen (*n* = 13+6)	2019	Hensley	US	VEN	IVC	10 pat. (surgery)	Supine vs prone	Diameter
	2011	Ho	US	VEN	IVC	24 vol.	Upright vs prone	Diameter
	2015	Kundra	US	VEN	IVC	32 pat. (term parturients)	Supine vs R-TB	Diameter
	2014	Panebianco	US	VEN	IVC	45 pat. (dialysis)	Supine vs R-TB (45°)	Diameter
	2001	Rist	US	VEN	CIV	10 pat. (surgery)	Supine vs TB (20°) vs R-TB (20°)	Diameter
	2010	Asbeutah	US	ART	CeA	30 vol.	Supine vs upright	Diameter
	2019	Krzanowski	US	VEN	CIV + LRV	41 pat. (w/ pelvic venous disorder)	Supine vs LL vs upright	CSA
	2020	Ebubedike	US	VEN	SV	82 pat. (w/ varicocele)	Supine vs upright	Diameter
	2021	Engelhorn	US	VEN	Left CIV	50 vol.	Prone vs upright	Diameter
	2016	Ukere	US	VEN	Right HV + PV	20 pat. (liver resection)	Supine vs R-TB (20°)	Diameter
	2021	Arshad	US	VEN	SV	35 pat. (w/ varicocele)	Supine vs upright	Diameter
	2011	Mookadam	US	VEN	IVC	43 pat. (w/ echocardiography)	Supine vs LL	Diameter
	2016	Najari	US	VEN	SV	114 pat. (w/ varicocele)	Supine vs upright	Diameter
	2020	Jinzaki	CT	A+V	VC + aorta	32 vol.	Supine vs upright	CSA
	2019	Kadoya	MRI	VEN	PV	12 vol.	Supine vs upright	CSA
	2021	Kadoya	MRI	A+V	IVC + AA	12 vol.	Supine vs upright	CSA
	2016	Saravanakumar	MRI	A+V	IVC + AA	6 pat. (obese and pregnant)	RL; LL; supine with pelvic tilt; R-TB (5°, 10° and 15°)	CSA
	2012	Ganesan	MRI	ART	CIA	7 pat. (MRI)	Supine vs prone	Vessel-spine distance
	2009	Choi	MRI	ART	Aorta + CIA	7 vol.	Supine vs fetal (hip flexion)	Curvature, length, angle, distance
Thorax (*n* = 7)	2019	Riccio	MRI	A+V	IVC + AA	20 vol.	Supine vs prone	Aorta-spine distance
	2015	Qian	MRI	ART	Aorta	22 pat. (w/ ankylosing spondylitis)	Supine vs prone	Aorta-spine distance
	2019	Plataniotis	CT	ART	Aorta	200 pat. (w/ lower back pain)	Supine vs prone	Aorta-spine distance
	2012	Jiang	MRI	ART	Aorta	26 pat. (w/ idiopathic scoliosis)	Supine vs prone	Aorta-spine distance
	2008	Yamaji	CT	VEN	PuV	116 pat. (w/ atrial fibrillation)	Supine vs prone	Diameter
	2019	Wieslander	MRI	A+V	PuV + PuA	10 vol.	Supine vs prone vs LL vs RL	CSA
	2021	Gottlieb	MRI	VEN	PuV	20 vol.	Supine vs LL	Diameter
Head-neck (*n* = 25 + 10)	2009	Morimoto	US	VEN	IJV + EJV + VV	22 vol.	Supine vs upright vs. TB (90°)	Diameter
	2016	Montero	US	ART	ICA + VA	10 vol.	TB (30°) vs supine vs R-TB (30°)	Diameter
	2012	Kantarci	US	VEN	IJV + VV	62 pat. (w/ multiple sclerosis) & 21 vol.	Supine vs upright	CSA
	2009	Saeed	US	ART	CCA	20 vol.	Supine vs upright (sitting)	Diameter
	2016	Yeoh	US	VEN	IJV	27 vol.	Supine vs prone vs RL	CSA
	2014	Lee	US	VEN	IJV	58 pat. (w/ ASA I-II)	Supine vs TB (10°)	Diameter, CSA
	2013	Jo	US	VEN	IJV	40 pat. (w/ ASA I-II)	Supine vs TB (10°)	CSA
	2019	Chen	US	VEN	IJV	82 pat. (prostatectomy)	Supine vs TB	CSA
	2004	Gisolf	US	VEN	IJV	6 vol.	Supine vs upright	CSA
	2017	Holmlund	US	VEN	IJV	17 vol.	Supine vs upright (0°, 16°, 71°)	CSA
	2019	Wang	US	VEN	IJV	20 vol.	Supine vs. upright (0°, with increment 10° till 90°)	CSA
	2008	Kim	US	VEN	IJV + SCVs	20 vol.	Supine vs. TB (15°) vs. R-TB (15°) vs. leg elevation (50°)	CSA
	2018	Judickas	US	VEN	IJV	63 vol.	30° R-TB, 45° passive leg raise, and 10° TB. All vs. Supine and with and without 30° head rotation	CSA
	2017	Watkins	US	VEN	IJV	15 vol.	Supine vs 15° head down tilt vs. sitting	CSA
	2021	Marshall-Goebel	US	VEN	IJV	10 vol.	Supine vs upright (sitting)	CSA
	2011	Mayer	US	VEN	IJV + VV	20 pat. (w/ multiple sclerosis) & 20 vol.	Supine vs upright (sitting)	CSA
	2019	Okamura	US	VEN	IJV	82 pat. (surgery)	Supine vs TB (10°)	CSA
	2019	Westlund	US	VEN	IJV	17 vol.	Supine vs upright (sitting, 0°, 16° and 71°)	CSA
	2019	Boschert	US	VEN	IJV + EJV	11 vol.	Supine vs TB (12°)	CSA
	2017	Holmlund	US	VEN	IJV	11 vol.	Supine vs. upright (8°, 16°, 24°, 32°, 40°, 69°)	CSA
	2004	Glor	US	ART	CCA (right)	9 vol.	Supine vs. supine with head in max. left turn	CSA
	2001	Muhammad	US	VEN	IJV	25 pat. (surgery)	Semi-prone (10° tilt) head positions: neutral vs lateral rotation (sideways) vs medial rotation (towards body)	Diameter
	2018	Park	US	ART	CCA	18 vol.	supine-head neutral vs supine-45° head rotation vs supine-80° head rotation vs LL vs RL	Distance from the C6 anterior tubercle to the carotid artery
	2021	Yu	US	ART	ICA	28 pat. (w/ ASA 1–11)	Supine vs TB	Diameter
	2011	Doepp	US	VEN	IJV + AV + VV	40 pat. (w/ multiple sclerosis)	Supine vs upright	CSA
	2013	Papaharilaou	MRI	ART	ICA + ECA + CCA	2 vol.	Supine vs. prone with up to 80° left and right head rotation	Angles between the ICA-CCA, ICA-ECA, and ECA-CCA
	2009	Aristokleous	MRI	ART	ICA + ECA + CCA	5 vol.	Supine vs. supine with up to 80° left and right head rotation	Angles between the ICA-CCA, the ICA-ECA, and ECA-CCA
	2011	Niggemann	MRI	VEN	IJV + EJV + NV + VP	15 vol.	Supine vs. upright (sitting)	Diameter
	2020	Kosugi	CT	A+V	IJV + EJV + ACV + ICA	20 vol.	Supine vs. upright	CSA
	2016	Holtackers	US + MRI	ART	CCA	12 vol.	Supine neutral vs. supine ~50° left head rotation + tilt back	Diameter, vessel length
	2017	Ishida	MRI	A+V	IJV + ICA + VA	22 vol.	Supine vs. –6° HDT vs. –12° HDT	CSA
	2022	van Zandwijk	MRI	VEN	IJV	15 vol.	Supine vs. 21°, 45°, 69° and 90° upright	Diameter
	2003	Vos	MRI	ART	CCA + ICA	7 pat. (carotid stenting)	5 different head positions (neutral, turned left and right, and bent forward and backward)	Artery angulation
	2017	Qureshi	DSA	A+V	IJV	3 pat. (angiography)	Supine vs. 60° upright	Diameter
	2012	Aristokleous	MRI	ART	CCA + ICA	2 vol.	Supine neutral vs. prone with 80° left head rotation vs. prone with 80° right rotation	Bifurcation angle, artery angle, CSA
Peripheral (*n* = 34 + 13)	2021	Mestre	US	VEN	SSV + DCV	57 pat. (w/ CVD) & 54 vol.	Supine vs upright (standing)	CSA
	2002	Lurie	US	VEN	SFV	10 pat. (w/ chronic venous insuff.)	Standing vs R-TB (15°)	CSA
	2012	DeMuth	US	VEN	GSV	28 pat (w/ venous insuff.)	Supine vs R-TB	Diameter
	2005	Groothuis	US	ART	CFA	11 pat (w/ spinal cord injury) & 10 vol.	Supine vs R-TB	Diameter
	2002	Hoballah	US	VEN	GSV	20 vol.	Supine vs R-TB (15°)	Diameter
	2003	Nguyen	US	VEN	FV	30 pat. (w/ gastric bypass)	Supine vs R-TB (30°)	CSA
	2013	Villar	US	ART	PoA	11 vol.	Supine vs TB (35°) vs R-TB (45°)	Diameter
	2003	Limpus	US	VEN	CFV	20 vol.	Sitting vs knee flexed (90°) vs LL vs supine	Diameter
	2005	Dix	US	VEN	PoV (and all deep veins)	29 pat. (w/ leg ulcers) & 10 vol.	Standing, sitting, horizontal, elevated leg with increment 5° till 45°	CSA
	2000	Delis	US	ART	PoA	36 pat. (w/ intermittent claudication) & 29 vol.	Recumbency, sitting, return to recumbency	Diameter
	2008	Newcomer	US	ART	BA + SFA	21 vol.	Supine vs seated vs standing	Diameter
	2019	Tauraginskii	US	VEN	GSV	61 pat. (w/ GSV incompetency)	Supine vs upright (w/ stretched legs) vs vertical	Diameter
	2019	Ciufo	US	VEN	PoV	13 vol.	Standing vs knee flexed	Diameter
	2006	Pemble	US	VEN	Superficial + deep leg veins	39 pat. (primigravida women)	Supine vs upright	CSA
	2013	Villar	US	ART	PoA	15 vol.	TB (35°) vs R-TB (45°)	Diameter
	2004	Delis	US	VEN	PoV + FV + CFV	13 vol.	Sitting vs standing	Diameter
	2006	Morita	US	VEN	PoV	21 vol.	Prone vs sitting	CSA
	2013	Warwick	US	VEN	SFV	10 vol.	Supine vs leg elevated vs upright	CSA
	2008	van Rij	US	VEN	FV	934 pat. (venous disease)	Standing vs sitting vs lying vs ambulating	Diameter
	2021	Reb	US	VEN	PoV	24 vol.	Upright straight leg vs knee flex. (crutch position, 90°)	Diameter
	2016	Lattimer	US	VEN	FV	11 pat. (obstruction group) & 11 pat. (reflux group) & 11 vol.	R-TB (70°) vs R-TB (45°) vs TB (40°)	Diameter
	2013	Villar	US	ART	PoA	15 vol.	Prone vs TB (35°) vs R-TB (45°)	Diameter
	2017	Becker	US	ART	ATA	18 vol.	Supine vs R-TB (15°, 6°) vs TB (6°, 15°)	Diameter
	2019	Yang	US	VEN	GSV + SSV	9 vol.	Supine or prone vs standing	Diameter
	2017	Villar	US	ART	PoA	10 vol.	Supine vs TB (35°) vs R-TB (45°)	Diameter
	2006	Cirovic	US	VEN	ATV + PTV + PeV + GSV + SSV	12 vol.	Supine, sitting legs horizontal, sitting one leg suspended, supine one leg raised	CSA
	2019	Claydon	US	ART	BA	16 vol.	Supine vs R-TB (60°)	Diameter
	2019	Brown	US	ART	PoA	45 pat. (15 w/ symptomatic lower extremity, 30 asymp.)	Ankle neutral vs maximally plantar flex.	Diameter
	2011	Levine	US	VEN	PoV	16 vol.	Supine vs knee flexed or hyperextended	Diameter
	2011	Pannucci	US	VEN	CFV	12 vol.	Supine w/ knee flex. (90°) & hip flex. (0°–30°–60°–90°)	Diameter
	2016	Sadek	US	VEN	SuV	49 vol.	Neutral vs arm abd. (90°)	CSA
	2009	Stapleton	US	ART	SuA	31 vol.	Arm in 12 positions (degrees of horizontal flex./ext., abd. and er.)	Diameter
	2007	Stapleton	US	ART	AA	26 vol.	Glenohumeral glide position	Diameter
	2006	Demondion	US	ART	SCA	28 pat. (w/ arterial TOS) & 44 vol.	Neutral vs abd. (90°, 130°, 17°)	CSA
	2013	Park	CT	ART	SCA	1 vol.	Supine vs a full-draw position in archery	CSA
	2020	Turin	MRI	VEN	GV (sup. + inf.)	16 vol.	Supine, prone, prone with a bump (jack-knife), lateral positions	Diameter, vessel location with respect to bony landmarks
	2021	Fujii	MRI	VEN	SSV + GSV	56 vol.	Supine vs sitting vs standing	CSA
	2021	Breen	MRI	ART	PoA	10 vol.	Supine w/ wedge again plantar side of feet vs supine w/ active plantar flex.	Diameter
	2010	Cheng	MRI	ART	SFA	7 vol.	Supine vs LL	Vessel length, axial twist, curvature
	2004	Diaz	DSA	ART	PoA	57 pat. (angiography)	Leg flex. (100°) vs extension	Distance between the popliteal hinch point and the medial supracondylar tubercle of the femur
	2013	Gökgöl	CT	ART	PoA	5 pat. (w/ symptomatic PAD)	Leg ext. vs flex. (70°, 20°) in the knee/hip	Vessel length, axial twist, curvature
	2017	Gökgöl	DSA	ART	SFA + PoA	35 pat. (w/ PAD)	Leg ext. vs flex. (70°, 20°) in the knee/hip	Vessel length, curvature
	2009	Klein	DSA	ART	SFA + PoA	9 pat. (w/ PAD)	Leg straight vs flex.	Vessel length, curvature, torsion
	2011	LaBan	CT	A+V	SCA + SCV	17 pat. (w/ TOS)	Arm neutral vs abd. (90°) w/ er.	Diameter
	2017	Rabtsun	CT + US	VEN	SFA + PoA	10 pat. (w/ SFA occlusion)	Leg straight vs flex. (110° hip, 20° knee)	Vessel length
	2019	Spinella	CT	ART	PoA + SFA	7 pat. (w/ popliteal aneurysm)	Straight leg vs 90° knee flex,	Vessel length, tortuosity index, curvature, diameter
	2004	Charon	MRI	A+V	SCA + SCV	51 pat. (w/ suspected TOS)	Arm abd. vs adducted	Diameter

*Acronyms: Modalities*: CT = computed tomography; DSA = digital subtraction angiography; MMOD = multiple modalities; MRI = magnetic resonance imaging; US = ultrasound. *System*: ART = arterial; VEN = venous; A+V = arterial and venous. *Subjects*: CVD = chronic venous disease; insuf. = insufficiency; pat. = patients. vol. = healthy volunteers. PAD = peripheral arterial disease; TOS = thoracic outlet syndrome. *Vessels*: AA = abdominal aorta; ACV = anterior condylar vein; ATA = anterior tibial artery; ATV = anterior tibial vein; AV = azygos vein; BA = Brachial artery; CCA = common carotid artery; CeA = celiac artery; CFA = common femoral artery; CFV = common femoral vein; CIA = common iliac artery; CIV = common iliac vein; DCV = deep calf vein; EJV = external jugular vein; FV = femoral vein; GSV = great saphenous vein; GV = gluteal veins; HV = hepatic vein; ICA = internal carotid artery; IJV = internal jugular vein; inf. = inferior; IVC = inferior vena cava; LRV = left renal vein; NV = nuchal veins; PeV = peroneal vein; PoA = popliteal artery; PoV = popliteal vein; PTV = posterior tibial vein; PuA = pulmonary artery; PuV = pulmonary vein; PV = portal vein; SCA = subclavian artery; SCV = subclavian vein; SFA = superficial femoral artery; SFV = superficial femoral vein; SSV = small saphenous vein; SuA = subclavian artery; sup. = superior; SuV = subclavian vein; SV = spermatic vein (also; varicocele vein / testicular vein); VA = vertebral artery; VC = vena cava; VP = veins of the vertebral plexus; VV = vertebral vein. *Positions*: abd. = abduction; ABER = abduction and external rotation; CL = contralateral; er. = external rotation; ex. = extension; flex. = flexion; HUT = head-up tilting; IL = ipsilateral; LDP = lateral decubitus position; LL = left lateral; R-TB = reverse Trendelenburg; RL = right lateral; TB = Trendelenburg; WB = weight bearing. *Outcomes*: CSA = cross-sectional area

### Abdomen

In the abdominal region, US was the most frequently reported imaging modality in different body postures and positions with 13 out of 19 articles [15–27]. Of these thirteen studies, the majority focused on the inferior vena cava (IVC) diameter that increased when going from supine to prone position by approximately 0.2 cm [15] and increased further with 0.2 cm from prone to standing position [27]. A 45° R-TB position did not influence the IVC diameter compared to supine position [16]. Interestingly, a left lateral position increased IVC diameters in a healthy population [17] and decreased diameters in elective cesarean section women [18]. Furthermore, the spermatic vein in patients with (suspected) varicocele increased in diameter in upright relative to supine position [19–21]. The common iliac vein diameter increased in R-TB [22] and upright position [23, 24]. For the right hepatic vein and portal vein dimensions, no correlation was found between supine and TB positions [26]. In the arterial system, only the celiac artery diameter was investigated for positional changes using US, which increased in standing position compared to supine [25].

MRI was used to study abdominal vasculature in five studies, mainly comparing upright and prone positions. The portal vein CSA decreased from supine to upright position [7]. Vena cava collapsibility was relatively high in upright position, whereas aorta CSA did not differ between supine and upright position at multiple aortic levels on MRI [28] and CT [6]. In obese pregnant women, R-TB position increased the aorta and IVC CSA by reducing vessel compression [29]. Two studies investigated common iliac artery diameters of which one (*n* = 97) did not find relevant differences in anatomical vascular orientation between prone and supine position [30], while the other (*n* = 7) observed shortening, higher curvature, and superior translation of the common iliac arteries in a hip flexion position [13].

### Thorax

Seven studies reported on position-dependent imaging of thoracic vasculature on either CT or MRI, of which five compared prone and supine position. The distance between the IVC and spine was smaller in prone position than in supine position [12]. The aorta-spine distances in patients with spine deformities were described to decrease by approx. 3 mm (*n* = 20, *n* = 200, and *n* = 26) [12, 31, 32] or stay equal (*n* = 22) [33] in prone position. The pulmonary veins showed increased CSA in prone position (patients with atrial fibrillation) and in lateral position (volunteers) [9, 34, 35], but CSA of pulmonary arteries was not affected by body position [34].

### Head-neck

The internal jugular vein (IJV) was extensively investigated using US in supine and upright positions, and its diameter and CSA were found to be around 0.90 cm^2^ in supine position and decreased or completely collapsed in upright position [36–46], which appeared to be more pronounced for the right IJV than the left IJV [37]. Similar results were found on the other imaging modalities MRI, CT, and DSA [47–49]. Other papers found that the IJV diameter and CSA increased up to 1.85 cm^2^ in TB compared to supine (*n* = 360) [41, 50–55], although this was not seen by Boschert et al [56] for both IJV and external jugular vein (*n* = 11). Additionally, the IJV CSA increased in prone position [57], with leg elevation [54] and with flexion of the head [58], while the IJV CSA decreased in R-TB position [54] and by rotating the head to the ipsilateral side [58], all compared to a neutral supine position. The external jugular vein and vertebral vein showed a diameter and CSA decrease in upright position compared to supine and TB positions [36, 37].

In the arterial system, the internal carotid artery (ICA) underwent no significant changes between supine and upright position on MRI [48]. According to Montero et al [59], the ICA and vertebral artery diameter increased in TB on US (*n* = 10), but this increase was not found by Yu et al (*n* = 28) [60]. No change in ICA or vertebral artery diameter was seen in R-TB position compared to supine [59]. For the common carotid artery, an upright position resulted in a diameter decrease from 6.7 to 6.5 mm relative to a supine position [61]. Maximal head rotation to the left had no effect on the right common carotid artery CSA compared to a neutral head position when investigated with US [62], while other MRI studies reported significant diameter change with head rotation, although without a specific tendency towards CSA increase or decrease [3, 63–66]. Holtackers et al [64] revealed that the difference between systolic and diastolic carotid artery diameters decreased when the volunteers’ heads were extended and rotated to the left. Ishida et al [67] investigated the influence of R-TB and TB on IJV, ICA, and vertebral artery CSA with MRI, where only the IJV diameter was found to significantly increase in TB compared to a supine position. Park et al [68] found that the distance between the common carotid artery and anterior C6 tubercle increased with increasing contralateral neck rotation.

### Peripheral

There were 34 articles in the peripheral category that used US as main imaging modality. Twenty-nine of these focused on lower-extremity vasculature in neutral supine position versus upright (standing), various degrees of TB and R-TB, hip flexion, knee flexion, elevated legs with multiple increments, sitting, and ankle plantar flexion [69–97].

The femoral vein diameter and CSA increased (up to higher than 50 mm^2^) in R-TB, knee flexion (sitting position), upright position, and hip flexion up to 60° compared to a supine position (approx. 28 mm^2^) [69–77]. The great saphenous vein, tibial vein, and small saphenous vein diameter and CSA increased in upright and R-TB positions compared to supine and prone positions [77–82, 88]. Only Yang et al [80] did not find measurable differences in tibial vein and small saphenous vein diameters in upright position compared to prone and supine positions (*n* = 9). Popliteal vein diameter and CSA were larger in upright or sitting positions than in supine and prone position, and in postures with leg elevation [72, 83, 84] or leg flexion [85], but did not differ between standing and kneeling positions [86, 87].

The anterior tibial artery diameter was not significantly different between supine and TB or R-TB positions [95], similar to the femoral artery diameter which did not differ between supine, seated, standing, and R-TB positions [96, 97]. Also, the popliteal artery did not show significant differences in diameter in TB or R-TB positions compared to supine or prone [89–93], but popliteal artery diameter did decrease with plantar ankle flexion because of calf muscle compression [94, 98]. Aside from US, seven studies investigating popliteal artery or superficial femoral artery using CT and angiography found that leg flexion shortens artery length up to 12% and increases curvature up to 100% [99–104]. On MRI, the superficial femoral artery shortened in the lateral position with leg flexion [105], and inferior and superior gluteal vein diameters decreased in lateral position on the contralateral side [106]. Furthermore, Fujii et al [107] found on MRI that the small saphenous vein CSA was significantly larger in the sitting and standing positions than in supine position.

In the upper extremity, six studies investigated the brachial artery [96, 108], axillary artery [109], subclavian artery [5, 110], and subclavian vein [111]. Different postures had no significant effect on brachial artery diameter [96, 108] or subclavian artery diameter and CSA in volunteers [110]. However, in subjects with (suspected) TOS, the subclavian artery CSA decreased or even occluded in a posture of combined head rotation with shoulder abduction and in abduction and exorotation investigated with CT and MRI [5, 112–114]. Furthermore, the axillary artery diameter decreased in a combined abduction, horizontal flexion, and external rotated posture [109].

## Discussion

This scoping review provided an overview of the currently available imaging modalities for visualization and characterization of human vascular anatomy in different body positions and postures. In this section, we will focus solely on the overall concept of different postures and positions rather than zooming in on specific body positions as the included literature covers a broad spectrum. Different postures (flexion, rotation, abduction, etc.) only make up 16% of the found papers, while different body positions (prone, upright, TB, etc.) were fairly well investigated (84%). The majority of the papers used US as imaging modality in different body positions and postures. Of these, a large portion investigated peripheral vasculature and found that the dimensions of lower-extremity veins decrease in positions with elevation of the feet. Similar observations of decreasing vein diameters were made for US examinations of the head and neck in head elevating positions. Diameter and CSA were the most commonly investigated outcome measures, which is related to a limited field of view in US that allows only for in-plane assessment of such measures [115]. Other modalities also allow assessment of geometric characteristics like vessel length, angulation, and distances between anatomical structures. This provides additional relevant information for clinical assessment that can be relevant in, for example, (positional) surgery planning [12].

Diameters and CSAs in the venous system appeared to be more sensitive to positional changes like upright, prone, R-TB/TB, and lateral positions than in the arterial. The difference between the venous and arterial positional deformation can be attributed to the structure of the vessel wall, which is more proliferated and thick for arteries than it is for veins. In our opinion, however, curvature and other geometrical parameters of the arterial and venous systems are similarly affected by postural changes such as flexion and rotation. With the increasing number of endovascular treatments of peripheral arterial occlusive disease and aortic aneurysms, it would be of interest to quantify the geometrical deformation of the target vessels so this can be taken into account in treatment planning and stent manufacturing. Geometrical deformation by positional and/or postural changes leads to different forces, torsion, or shear stresses along a vessel and the in situ stents [116]. This would especially be of interest in arteries (and veins) in body parts that allow for more movement, for example, the femoral and popliteal arteries during leg and hip flexion, but also the carotid, vertebral, and axillary arteries during head rotation. Characterizing the deformation will aid in optimization of stent placement and identification, and thereby reduce the risk for complications such as stent kinking and fractures [13, 65, 75]. Moreover, the deformation of these structures in different postures could be used as input for patient-specific computational fluid dynamics analysis to identify deviating flow patterns that could lead to adverse events [117]. Position-dependent imaging could provide essential information in treatment procedures such as spine surgery [12, 31–33] or pulmonary vein isolation therapy [9, 34, 35].

The search results in this review also held papers discussing several syndromes that relate to an aberrant vascular anatomy due to body positions and postures, such as TOS and Bow Hunter’s syndrome. Since most of these syndromes are fairly rare and presented as case reports and/or without proper outcome measures, they may need independent consideration. Bow Hunter’s syndrome studies were excluded from the present review considering the large number of papers available regarding this syndrome and already available reviews [10, 11]. In this syndrome, neck rotation or extension causes mechanical compression of the vertebral artery, most commonly caused by an osteophyte. All imaging modalities that were discussed in this review are widely used in the diagnosis and evaluation of this syndrome, with digital subtraction angiography considered as the gold standard. Nonetheless, both TOS and Bow Hunter’s syndrome are important examples of how different postures can affect vessel geometry.

Subjects in the included studies were predominantly healthy volunteers or patients scheduled for diagnostic imaging or (elective) surgery that was not related to any vascular pathology. Even though the results of these studies give a proper indication of the vessel deformation in different positions and postures, it should be taken into account that the behavior of diseased vessels may differ when the treatment plan is determined. Furthermore, based on the articles that were evaluated in this review, a wide and heterogeneous range of clinical purposes was observed that relate the vessel deformation to diagnosis or treatment outcome. Physicians should be aware that the orientation in which vessels are being imaged in the preprocedural phase is not representative of the dynamic forces during everyday movement. The vessels undergo ever-changing deformation due to the different positions and postures a person adopts during the day.

### Limitations

The present review focused on anatomical and geometrical deformation of vasculature in different body positions or postures and did not evaluate functional parameters such as blood flow measurements with duplex US. In clinical practice, evaluation of such functional parameters may be relevant as well. A separate review on functional vascular imaging in different body positions and postures may be advised.

## Conclusion

Vascular geometry in different body positions and postures was predominately evaluated with the outcome measures vessel diameter and CSA using US as imaging modality. Positional changes were more often evaluated than postural changes. Venous diameters and CSA were generally more sensitive to positional changes like upright, prone, R-TB/TB, and lateral positions than the arterial equivalents. However, curvature and other geometrical parameters of the arterial and venous systems are equally affected by postural changes (e.g., flexion, abduction, rotation), which was often evaluated on CT or MRI, rather than US. The most important clinical implications of positional changes are found in diagnosis, surgical planning, and in the stent placement and follow-up. However, the knowledge of the influence of body positions and postures on the vasculature and how these may affect treatment of vascular pathologies remains limited, such as the influence of bending of the knee on stent geometry in atherosclerotic popliteal arteries.

### Supplementary information

ESM 1 (XML 3 kb)

## Abbreviations

A+V: Arterial and venous
AA: Abdominal aorta
abd.: Abduction
ABER: Abduction and external rotation
ACV: Anterior condylar vein
ART: Arterial
ASA: American Society of Anesthesiology classification score
ATA: Anterior tibial artery
ATV: Anterior tibial vein
AV: Azygos vein
BA: Brachial artery
CCA: Common carotid artery
CeA: Celiac artery
CFA: Common femoral artery
CFV: Common femoral vein
CIA: Common iliac artery
CIV: Common iliac vein
CL: Contralateral
CSA: Cross-sectional area
CT: Computed tomography
CTA: Computed tomography angiography
CVD: Chronic venous disease
DCV: Deep calf vein
DSA: Digital subtraction angiography
EJV: External jugular vein
er.: External rotation
ex.: Extension
flex.: Flexion
FV: Femoral vein
GSV: Great saphenous vein
GV: Gluteal veins
HUT: Head-up tilting
HV: Hepatic vein
ICA: Internal carotid artery
IJV: Internal jugular vein
IL: Ipsilateral
inf.: Inferior
insuf.: Insufficiency
IVC: Inferior vena cava
LDP: Lateral decubitus position
LL: Left lateral
LRV: Left renal vein
MMOD: Multiple modalities
MRA: Magnetic resonance angiography
MRI: Magnetic resonance imaging
NV: Nuchal veins
PAD: Peripheral arterial disease
pat.: Patients
PeV: Peroneal vein
PoA: Popliteal artery
PoV: Popliteal vein
PTV: Posterior tibial vein
PuA: Pulmonary artery
PuV: Pulmonary vein
PV: Portal vein
RL: Right lateral
R-TB: Reverse Trendelenburg
SCA: Subclavian artery
SCV: Subclavian vein
SFV: Superficial femoral vein
SSV: Small saphenous vein
SuA: Subclavian artery
sup.: Superior
SuV: Subclavian vein
SV: Spermatic vein (also varicocele vein/testicular vein)
TB: Trendelenburg
TOS: Thoracic outlet syndrome
US: Ultrasound
VAa: Vertebral artery
VC: Vena cava
VEN: Venous
vol.: Healthy volunteers
VP: Veins of the vertebral plexus
VV: Vertebral vein
WB: Weight bearing

## Declarations

### Guarantor

The scientific guarantor of this publication is Prof. MD Robert H. Geelkerken.

### Statistics and biometry

No complex statistical methods were necessary for this paper.

### Study subjects or cohorts overlap

All study subjects and cohorts have been previously reported since this is a review.

### Methodology

- Performed at one institution
