# Supplementary material for: Position- and posture-dependent vascular imaging—a scoping review
Source: Eur Radiol. 2023 Sep 6;34(4):2334–51. doi: 10.1007/s00330-023-10154-9 (PMC10957623; doi:10.1007/s00330-023-10154-9)
Supplement: Supplementary file 1 — (XML 3 kb) [file 330_2023_10154_MOESM1_ESM.pdf]

Electronic supplementary material (ESM)

Search inputs for the following databases were:

Scopus: (TITLE-ABS(angiograph* OR CT OR CTA OR "computed tomography" OR MRI OR MRA OR "magnetic resonance imaging" OR MRV OR "magnetic resonance venography" OR "US imag*" OR "ultrasound" OR "medical imaging" OR fluoroscop* OR DSA OR nuclear OR radiograph* OR "x-ray") AND TITLE-ABS(vascular OR "blood vessel" OR arter* OR venous OR vein* OR aort* OR angiograph* OR venograph* OR "vena cava" OR vessel*) AND TITLE-ABS((upright OR "sedentary position" OR recumbent OR "prone position" OR lotus OR "cross-legged" OR Trendelenburg OR posture OR "position dependent" OR "body position") OR ((limb OR elbow OR head OR neck OR shoulder OR leg OR arm OR wrist OR spine OR hip OR knee OR thorax OR abdomen OR pelvis OR extremit*) AND (flex* OR extens* OR *rotat* OR abduct* OR adduct* OR supinat* OR pronat* OR inversion OR eversion))) AND TITLE-ABS(geometry OR tortuous* OR curv* OR diameter OR "cross-sectional" OR pattern* OR drain* OR metric* OR rotat* OR angulat*)) AND PUBYEAR > 1999 AND LANGUAGE(English)

Medline: ((angiograph*[Title/Abstract] OR CT [Title/Abstract] OR CTA [Title/Abstract] OR "computed tomography"[Title/Abstract] OR MRI [Title/Abstract] OR MRA [Title/Abstract] OR "magnetic resonance imaging"[Title/Abstract] OR MRV [Title/Abstract] OR "magnetic resonance venography"[Title/Abstract] OR "US imag*"[Title/Abstract] OR "ultrasound"[Title/Abstract] OR "medical imaging"[Title/Abstract] OR fluoroscop*[Title/Abstract] OR DSA[Title/Abstract] OR nuclear[Title/Abstract] OR radiograph*[Title/Abstract] OR "x-ray"[Title/Abstract]) AND (vascular[Title/Abstract] OR "blood vessel"[Title/Abstract] OR arter*[Title/Abstract] OR venous[Title/Abstract] OR vein*[Title/Abstract] OR aort*[Title/Abstract] OR angiograph*[Title/Abstract] OR venograph*[Title/Abstract] OR "vena cava"[Title/Abstract] OR vessel*[Title/Abstract]) AND ((upright[Title/Abstract] OR "sedentary position"[Title/Abstract] OR recumbent[Title/Abstract] OR "prone position"[Title/Abstract] OR lotus[Title/Abstract] OR "cross-legged"[Title/Abstract] OR Trendelenburg[Title/Abstract] OR posture[Title/Abstract] OR "position dependent"[Title/Abstract] OR "body position"[Title/Abstract]) OR ((limb[Title/Abstract] OR elbow[Title/Abstract] OR head[Title/Abstract] OR neck[Title/Abstract] OR shoulder[Title/Abstract] OR leg[Title/Abstract] OR arm[Title/Abstract] OR wrist[Title/Abstract] OR spine[Title/Abstract] OR hip[Title/Abstract] OR knee[Title/Abstract] OR thorax[Title/Abstract] OR abdomen[Title/Abstract] OR pelvis[Title/Abstract] OR extremit*[Title/Abstract]) AND (flex*[Title/Abstract] OR extens*[Title/Abstract] OR *rotat*[Title/Abstract] OR abduct*[Title/Abstract] OR adduct*[Title/Abstract]OR supinat*[Title/Abstract] OR pronat*[Title/Abstract] OR inversion[Title/Abstract] OR eversion[Title/Abstract]))) AND (geometry[Title/Abstract] OR tortuous*[Title/Abstract] OR curv*[Title/Abstract] OR diameter[Title/Abstract] OR "cross-sectional"[Title/Abstract] OR pattern*[Title/Abstract] OR drain*[Title/Abstract] OR metric*[Title/Abstract] OR rotat*[Title/Abstract]OR "angulat*"[Title/Abstract])) AND ((2000/1/1:2022/06/30 [pdat]) AND (english[Filter]))

Cochrane: (("angiograph*" OR "CT" OR "CTA" OR "computed tomography" OR "MRI" OR "MRA" OR "magnetic resonance imaging" OR MRV OR "magnetic resonance venography" OR "US imag*" OR "ultrasound" OR "duplex" OR "medical imaging") AND ("vascular" OR "blood vessel" OR arter* OR venous OR vein* OR aort* OR "angiograph*" OR venograph* OR "vena cava") AND (upright OR "sedentary position" OR "recumbent" OR "prone position" OR lotus OR cross-legged OR Trendelenburg OR posture OR "position dependent" OR "body position") AND (advantage OR value OR improvement OR change* OR increase OR decrease OR geometry OR tortuous* OR curv* OR

diameter OR "cross-sectional" OR benefit OR pattern* OR drainage OR "diagnostic potential" OR "metric*")):ti,ab,kw" with Cochrane Library publication date Between Dec 1999 and Aug 2022

SET limits: Cochrane Library Publication Date: Between Dec 1999 – Jul 2022
